# 左旋门冬酰胺酶对伯基特淋巴瘤细胞株增殖、细胞周期及凋亡的影响

**DOI:** 10.3760/cma.j.issn.0253-2727.2021.11.008

**Published:** 2021-11

**Authors:** 冬云 涂, 猛 张, 纹静 尹, 林艳 徐, 威 桑, 振宇 李, 开林 徐

**Affiliations:** 1 徐州医科大学血液病研究所，徐州医科大学附属医院细胞研究和转化医学中心，徐州医科大学附属医院血液科，徐州 221000 Institute of Hematology, Xuzhou Medical University, Cell Research and Transformation Center, Affiliated Hospital of Xuzhou Medical University, Department of Hematology, Affiliated Hospital of Xuzhou Medical University, Xuzhou 221000, China; 2 南京中医药大学附属盐城市中医院心内科，盐城 224000 Department of Cardiology, Yancheng TCM Hospital Affiliated to Nanjing University of Chinese Medicine, Yancheng 224000, China

**Keywords:** 伯基特淋巴瘤, 左旋门冬酰胺酶, 细胞凋亡, Burkitt lymphoma, L-asparaginase, Apoptosis

## Abstract

**目的:**

探讨左旋门冬酰胺酶对伯基特淋巴瘤细胞株增殖、细胞周期和凋亡的影响及其作用机制。

**方法:**

应用CCK-8法检测左旋门冬酰胺酶对伯基特淋巴瘤细胞株细胞增殖的影响，流式细胞术检测细胞凋亡率和细胞周期，实时定量PCR和Western blot检测分析细胞周期、凋亡、自噬和PI3K/Akt/mTOR信号通路中各种分子的表达变化。

**结果:**

左旋门冬酰胺酶明显抑制多种伯基特淋巴瘤细胞株的增殖，并引起细胞周期G_0_/G_1_期阻滞，诱导细胞凋亡和自噬。进一步结果表明左旋门冬酰胺酶抑制c-Myc的表达，同时抑制p-PI3K、p-Akt-S473、p-mTOR、p-70S6K和p-4E-BP1的表达。PI3K抑制剂LY294002联合左旋门冬酰胺酶进一步诱导了细胞凋亡。此外，左旋门冬酰胺酶抑制STAT和ERK信号通路。

**结论:**

左旋门冬酰胺酶抑制伯基特淋巴瘤细胞株细胞增殖和G_0_/G_1_期细胞阻滞，诱导自噬和凋亡并通过抑制PI3K/Akt/mTOR信号通路调控细胞凋亡。

## Abstract (en)

**Objective:**

To investigate the effect of L-asparaginase on the proliferation, cell cycle, and apoptosis of Burkitt lymphoma cell lines and explore the molecular mechanism.

**Methods:**

The effect of L-asparaginase on the cell proliferation of Burkitt lymphoma cell lines was detected using the CCK-8 method. The apoptosis rate and cell cycle were detected using flow cytometry. The expression of related molecules in cell cycle, apoptosis, autophagy, and PI3K/Akt/mTOR signaling pathway was detected and analyzed using qPCR and Western blot assay.

**Results:**

L-asparaginase significantly inhibited the proliferation of Burkitt lymphoma cell lines and caused cell cycle arrest at G_0_/G_1_ phage. L-asparaginase induced cell apoptosis and autophagy in Burkitt lymphoma cell lines. Further results showed that L-asparaginase inhibited the expression of c-Myc and also inhibited the expression of p-PI3K, p-Akt-S473, p-mTOR, p-70S6K, and p-4E-BP1. Combining PI3K inhibitor LY294002 with L-asparaginase further induced apoptosis. Additionally, L-Asp inhibited STAT and ERK signaling pathways.

**Conclusion:**

L-asparaginase inhibited Burkitt lymphoma cell proliferation, arrested cell cycle, activated autophagy, and induced apoptosis by inhibiting the PI3K/Akt/mTOR signaling pathway.

伯基特淋巴瘤（Burkitt lymphoma，BL）是一种高度侵袭性B细胞非霍奇金淋巴瘤（NHL）。根据临床和生物学特征，BL分为三种亚型：地方型、散发型和免疫缺陷相关型[1]。地方型流行于非洲，90％以上与EB病毒（EBV）感染相关；散发型最为常见，15％左右与EBV相关；在免疫功能低下的患者中，BL主要与人类免疫缺陷病毒（HIV）感染相关，其中40％～50％ EBV阳性[2]。目前短疗程、高强度的化疗方案对此病治疗效果尚可，但化疗相关不良反应在中老年人和免疫抑制患者中十分显著[3]–[4]。部分BL患者，尤其是EBV阳性患者，仍然存在较高的难治复发风险[5]。因此有必要开发不良反应较小而治愈率较高的治疗方案。

阻断氨基酸合成的抗代谢药物已被开发并应用于多种肿瘤治疗[6]–[7]，其中左旋门冬酰胺酶（L-Asp）可以耗竭天门冬酰胺和谷氨酰胺[8]，是治疗急性淋巴细胞白血病（ALL）、NK/T细胞淋巴瘤（NKTCL）等血液系统恶性肿瘤的核心化疗药物[9]–[11]。流行病学数据表明，NKTCL的发病与EBV感染密切相关[12]。鉴于BL和NKTCL都是与EBV高度相关的淋巴瘤，我们尝试探索L-Asp对BL细胞株的作用，并对其相关机制进行研究。

BL的特征是c-Myc基因的易位和重排，导致c-Myc的结构性过表达[13]。MYC基因家族通过改变生物能量及代谢激活原癌基因并抑制抑癌基因，调控细胞增殖[14]。既往研究已经确定PI3K信号是成熟B细胞中B细胞受体（BCR）介导的生存信号[15]，PI3K信号可以通过阻断c-Myc的降解维持c-Myc的活性[16]–[17]。ERK信号通路与多种细胞活动有关，包括细胞增殖、分化、存活、死亡和转化[18]。信号转导和转录激活因子3（STAT3）是Janus激酶（JAK）/STAT信号通路中重要且研究最多的转录因子[19]。肿瘤细胞常存在STAT3蛋白的异常持续活化，与淋巴造血组织肿瘤的关系尤为密切[20]。综上，我们进一步探索了L-Asp对BL细胞株c-Myc表达、PI3K/Akt/mTOR信号通路、ERK信号通路、STAT3信号通路的影响。

## 材料与方法

1. 试剂：L-Asp购自中国广东广州明星药业有限公司，IMDM培养液购自美国Hyclone公司，胎牛血清（FBS）购自美国Gibco公司，二甲基亚砜（DMSO）购自美国Sigma公司，CCK-8试剂盒为日本同仁化学研究所产品。Annexin Ⅴ/7-AAD试剂盒为美国Becton Dickinson公司产品，碘化丙啶（propidium iodide, PI）购自美国Sigma公司，抗体P21、P27、DR5、Caspase-8、Caspase-3、PARP、Mcl-1、XIAP、Bax、Bim、Noxa、Cytochrome C、LC3B、ATG7、p-PI3K、p-Akt-S473、Akt、p-mTOR、p-70S6K、p-4E-BP1、p-STAT3、STAT3、p-ERK、ERK、ACTB均购自美国Cell Signaling Technology公司，辣根过氧化物酶标记二抗购自美国Bio-Rad公司。

2. 细胞培养：人BL细胞株Raji、Namalwa和CA46用含10％FBS的IMDM培养液，于37 °C、5％CO_2_、饱和湿度的培养箱中培养，每日于电子显微镜下观察细胞的生长状态，2～3 d按1∶3的比例进行传代培养。

3. CCK-8法检测细胞增殖：将密度2×10^4^/ml的细胞悬液按100 µl/孔接种于96孔板，处理组每孔加入指定浓度的L-Asp，同时设立空白组和阴性对照组，每组设3个复孔，培养24 h或48 h。每孔加入10 µl CCK-8溶液，在培养箱内孵育2 h，用酶标仪测定450 nm处的吸光度（*A*_450_）值。本实验设空白组、阴性对照组、药物处理组。按下列公式计算细胞增殖率：细胞增殖率（％）=［（药物处理组*A*_450_−空白组*A*_450_）/（阴性对照组*A*_450_−空白组*A*_450_）］×100％

4. 细胞周期的流式细胞术检测：取对数生长期的细胞接种于12孔板中（每孔1×10^6^个细胞），分为对照组和L-Asp处理组，24 h后收集细胞，计数1×10^6^细胞，用PBS洗细胞2遍，70％乙醇于−20 °C固定过夜，次日取出，用PBS洗细胞，加入300 µl PBS重悬细胞，加入5 µl 1 mg/ml PI和1 µl 1mg/ml RNA酶，避光孵育15 min，流式细胞术检测细胞周期，并用ModFit LT V3.3软件分析。

5. 细胞凋亡的流式细胞术检测：取对数生长期的细胞接种于12孔板中（每孔1×10^6^个细胞），分为对照组和L-Asp处理组，48 h后收集细胞，用PBS洗涤2遍，再用l×binding buffer缓冲液制成1×10^6^/ml细胞悬液。流式细胞管内加入100 µl细胞悬液，加入5 µl AnnexinⅤ-PE和5 µl 7-AAD，轻轻摇匀，室温避光染色15 min，加入1×binding buffer缓冲液200 µl，30 min内上流式细胞仪检测。

6. Western blot检测蛋白表达：收集细胞沉淀，加入适量细胞裂解液，离心收集上清，应用BCA法测定蛋白浓度，进行蛋白定量。各取50 µg总蛋白进行SDS-PAGE电泳，将蛋白样品转移到PVDF膜，5％脱脂奶粉室温慢摇，封闭1 h。弃去封闭液，一抗孵育过夜，二抗室温孵育1 h。使用磷酸盐吐温缓冲液（PBST）洗涤3次后加入适量ECL底物，在Image Quant LAS400 mini下扫描，曝光成像。

7. RNA提取和实时定量PCR：将人BL细胞株接种在6孔板中（每孔2×10^6^个细胞），并用指定浓度的L-Asp处理48 h。收集细胞，根据说明书使用TRIzol™试剂（美国Life Technologies公司产品）提取RNA。使用NanoDrop检测RNA浓度，然后使用M-MLV逆转录酶（美国Life Technologies公司产品）将RNA逆转录为cDNA。根据说明书，使用LightCycler480 SYBR^®^ Green Supermix（美国Roche公司产品）进行定量聚合酶链反应（qPCR）。用于实时荧光qPCR（RT-qPCR）基因的引物序列见表1。PCR扩增程序如下：94 °C 2 min，94 °C 30 s，60 °C 35个循环30 s，72 °C 35 s，最后在72 °C延伸2 min。采用2^−ΔΔCT^法计算目标基因的阈值循环数（Ct）和相对mRNA表达量。ACTB用作内部对照。

**表1:** 实时荧光定量PCR引物序列

基因名称	引物序列
Cyclin E	5′-TTCTTGAGCAACACCCTCTTCTGCAGCC-3′（正向引物） 5′-TCGCCATATACCGGTCAAAGAAATCTTGTGCC-3′（反向引物）
P21	5′-TGAGCCGCGACTGTGATG-3′（正向引物） 5′-GTCTCGGTGACAAAGTCGAAGTT-3′（反向引物）
P27	5′-TGCAACCGACGATTCTTCTACTCAA-3′（正向引物） 5′-CAAGCAGTGATGTATCTGATAAACAAGGA-3′（反向引物）
LC3B	5′-CGCACCTTCGAACAAAGAG-3′（正向引物） 5′-CTCACCCTTGTATCGTTCTATTATCA-3′（反向引物）
ATG7	5′-ACCCAGAAGAAGCTGAACGA-3′（正向引物） 5′-CTCATTTGCTGCTTGTTCCA-3′（反向引物）
ATG5	5′-TTTGAATATGAAGGCACACC-3′（正向引物） 5′-TGCAATCCCATCCAGAGTTG-3′（反向引物）
ACTB	5′-CTCCATCCTGGCCTCGCTGT-3′（正向引物） 5′-GCTGTCACCTTCACCGTTCC-3′（反向引物）

8. 统计学处理：采用GraphPad Prism 8软件对数据进行统计学分析，数据用*x±s*表示，多样本均数的比较采用单因素方差分析，*P*<0.05表示差异有统计学意义。

## 结果

1. L-Asp抑制BL细胞株增殖：L-Asp作用48 h后，在>0.1 U/ml的浓度下对Raji、Namalwa和CA46细胞的增殖有明显的抑制作用（图1），其抑制率在0.1～1 U/ml的浓度区间内呈剂量依赖关系，L-Asp作用48 h的抑制率高于24 h（图2）。

**图1:**
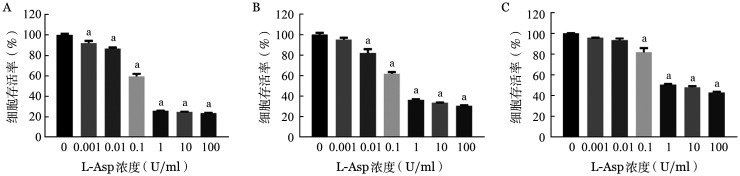
CCK-8法检测左旋门冬酰胺酶（L-Asp）对Raji（A）、Namalwa（B）和CA46（C）细胞增殖活性的影响（实验重复3次） a：与L-Asp浓度为0 U/ml组相比，*P*<0.001

**图2:**
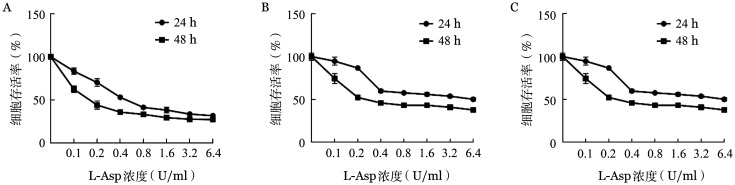
CCK-8法检测左旋门冬酰胺酶（L-Asp）作用24 h、48 h对Raji（A）、Namalwa（B）和CA46（C）细胞增殖活性的影响（实验重复3次）

2. L-Asp诱导BL细胞株发生G_0_/G_1_期阻滞：PI单染流式细胞术显示，0、0.4、0.8 U/ml的L-Asp作用于Raji、Namalwa和CA46细胞24 h后，与对照组相比，G_0_/G_1_期细胞增加，S期细胞减少，发生G_0_/G_1_期阻滞（图3）。RT-qPCR结果显示P21和P27的 mRNA 水平升高，Cyclin E的 mRNA水平降低（图4）。Western blot结果同样证实L-Asp使P21和P27的表达增加（图5）。

**图3:**
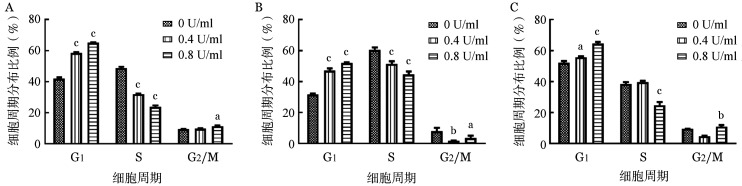
流式细胞术检测左旋门冬酰胺酶（L-Asp）对Raji（A）、Namalwa（B）和CA46（C）细胞周期的影响（实验重复3次） 与L-Asp浓度为0 U/ml组相比，^a^*P*<0.05，^b^*P*<0.01，^c^*P*<0.001

**图4:**
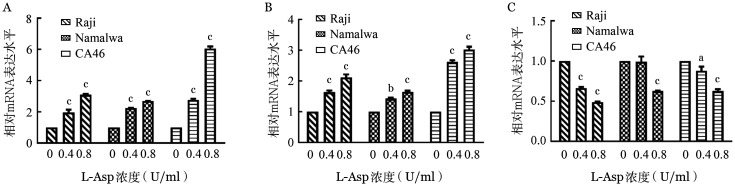
实时荧光PCR检测左旋门冬酰胺酶（L-Asp）对Raji、Namalwa和CA46细胞P21（A）、P27（B）、Cyclin E（C）mRNA表达水平的影响（实验重复3次） 与L-Asp浓度为0 U/ml组相比，^a^*P*<0.05，^b^*P*<0.01，^c^*P*<0.001

**图5:**
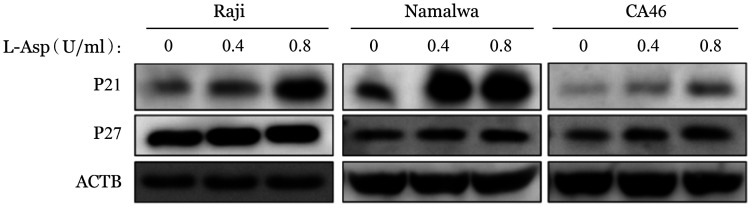
Western blot法检测左旋门冬酰胺酶（L-Asp）对Raji、Namalwa和CA46细胞P21、P27表达的影响 ACTB：内部对照

3. L-Asp诱导BL细胞株细胞凋亡：Annexin Ⅴ-PE和7-AAD双染流式细胞术显示，在Raji、Namalwa和CA46细胞中，0、0.4、0.8 U/ml的L-Asp作用48 h后，与对照组相比，凋亡细胞占比明显增多，提示L-Asp诱导BL细胞株细胞凋亡（图6）。

**图6:**
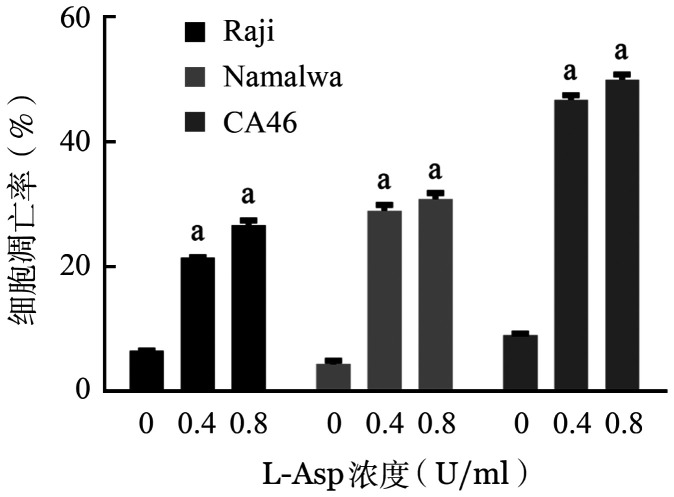
流式细胞术检测左旋门冬酰胺酶（L-Asp）对Raji、Namalwa和CA46细胞凋亡的影响（实验重复3次） 与L-Asp浓度为0 U/ml组相比，^a^*P*<0.001

4. L-Asp激活BL细胞株外源性细胞凋亡通路：Western blot结果显示，L-Asp可以诱导死亡受体5（DR5）上调，也可以诱导Caspase-8发生活化，进而诱导下游效应蛋白Caspase-3切割活化，其底物 PARP水解作用增强（图7）。

**图7:**
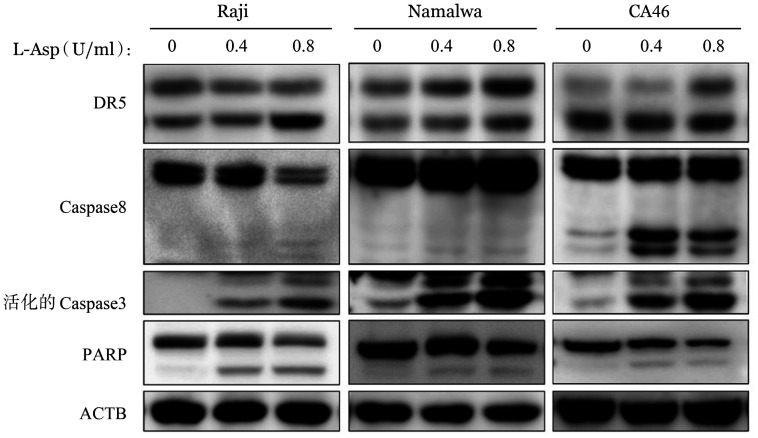
Western blot法检测左旋门冬酰胺酶（L-Asp）对Raji、Namalwa和CA46细胞外源性细胞凋亡通路的影响 ACTB：内部对照

5. L-Asp激活BL细胞株内源性细胞凋亡通路：检测L-Asp处理前后BL细胞株中Bcl-2家族相关蛋白的表达情况。结果显示，与对照组相比，实验组的促凋亡蛋白Bax、Bim、Noxa表达升高，而抗凋亡蛋白Mcl-1、XIAP表达降低，电子转运蛋白Cytochrome C表达升高（图8）。

**图8:**
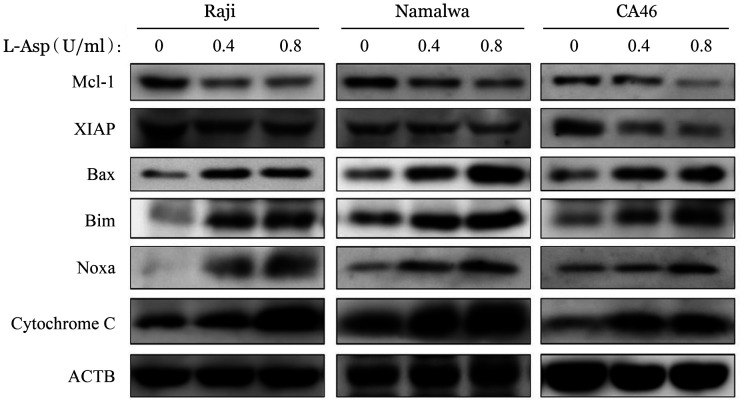
Western blot法检测左旋门冬酰胺酶（L-Asp）对Raji、Namalwa和CA46细胞内源性细胞凋亡通路的影响 ACTB：内部对照

6. L-Asp引起BL细胞株自噬：用指定浓度的L-Asp处理三种细胞株48 h后，通过RT-qPCR检测自噬标志物如ATG5、ATG7、LC3B，发现其mRNA水平明显升高（图9）。通过Western blot法检测自噬相关蛋白的表达，显示BL细胞株中 LC3B、ATG7的蛋白质水平也增加（图10）。

**图9:**
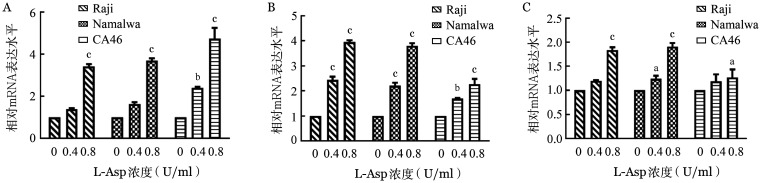
实时荧光定量PCR检测左旋门冬酰胺酶（L-Asp）对Raji、Namalwa和CA46细胞LC3B（A）、ATG5（B）、ATG7（C）mRNA水平的影响 与L-Asp浓度为0 U/ml组相比，^a^*P*<0.05，^b^*P*<0.01，^c^*P*<0.001

**图10:**
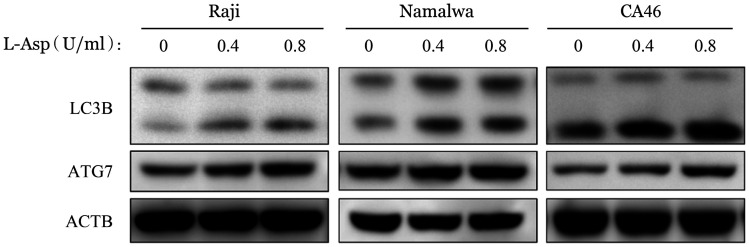
Western blot法检测左旋门冬酰胺酶（L-Asp）对Raji、Namalwa和CA46细胞自噬相关蛋白表达水平的影响 ACTB：内部对照

7. L-Asp抑制BL细胞株c-Myc的表达：用L-Asp处理 BL细胞株48 h后，细胞中c-Myc的蛋白质水平降低（图11），表明L-Asp抑制BL细胞株中c-Myc的表达。

**图11:**
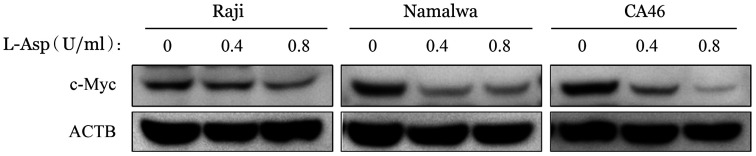
Western blot法检测左旋门冬酰胺酶（L-Asp）对Raji、Namalwa和CA46细胞c-Myc表达水平的影响 ACTB：内部对照

8. L-Asp抑制BL细胞株PI3K/Akt/mTOR信号通路：Western blot结果显示，L-Asp处理后，BL细胞株中p-PI3K、p-Akt-S473、p-mTOR、p-70S6K和P-4E-BP1的表达均明显降低，Akt未见明显变化，说明L-Asp可抑制BL细胞PI3K/Akt/mTOR信号通路的活化（图12）。

**图12:**
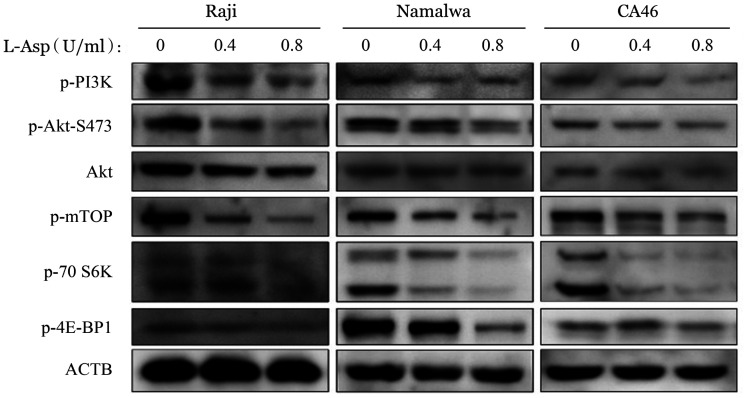
Western blot法检测左旋门冬酰胺酶（L-Asp）对Raji、Namalwa和CA46细胞PI3K/Akt/mTOR信号通路的影响 ACTB：内部对照

为进一步验证L-Asp对PI3K/Akt/mTOR信号通路的调控，我们将PI3K抑制剂LY294002与L-Asp 联合应用。当用L-Asp（0.4 U/ml）联合LY294002（20 µmol/L）处理BL细胞株时，p-Akt-S473和p-mTOR的水平均进一步降低（图13）。Annexin Ⅴ-PE和7-AAD双染流式细胞术检测结果显示，与单独应用L-Asp或 LY294002相比，联合用药组细胞凋亡明显增加（图14）。

**图13:**
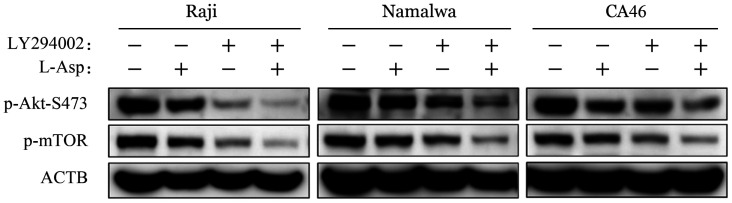
Western blot法检测左旋门冬酰胺酶（L-Asp）和PI3K抑制剂LY294002对Raji、Namalwa和CA46细胞PI3K/Akt/mTOR信号通路的影响

**图14:**
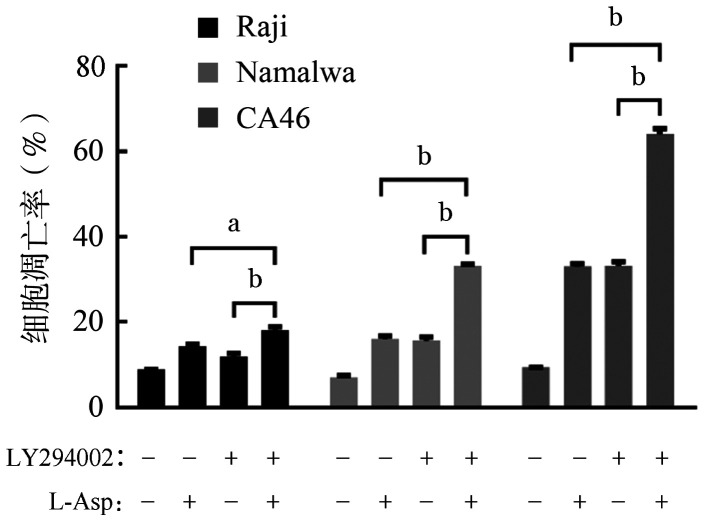
流式细胞术检测左旋门冬酰胺酶（L-Asp）和PI3K抑制剂LY294002对Raji、Namalwa和CA46细胞凋亡的影响（实验重复3次） ^a^*P*<0.05，^b^*P*<0.001

9. L-Asp对BL细胞株中ERK信号通路的影响：p-ERK的表达水平在L-Asp作用后降低，ERK的表达无明显变化（图15），说明L-Asp可以抑制BL细胞中ERK信号通路的活化。

**图15:**
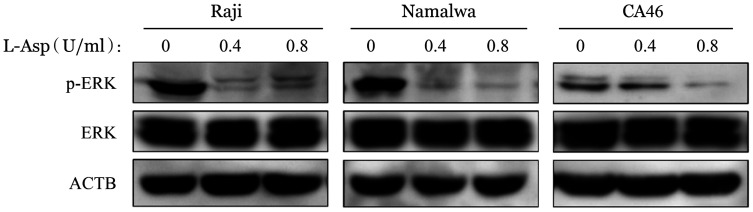
Western blot法检测左旋门冬酰胺酶（L-Asp）对Raji、Namalwa和CA46细胞ERK信号通路的影响

10. L-Asp对BL细胞株STAT3信号通路的影响：BL细胞STAT3磷酸化（p-STAT3）水平在L-Asp处理后降低，STAT3为见明显变化（图16），表明BL细胞中STAT3信号通路的活化受到了L-Asp的抑制。

**图16:**
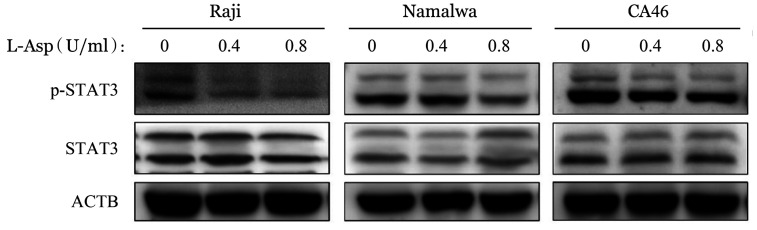
Western blot法检测左旋门冬酰胺酶（L-Asp）对Raji、Namalwa和CA46细胞STAT3信号通路的影响

## 讨论

L-Asp作为一种有效的抗肿瘤细胞蛋白质合成抑制剂，在治疗多种血液肿瘤，特别是ALL和NKTCL中作用显著。然而，L-Asp对BL的作用及其机制尚未完全阐明。在本研究中，我们研究了L-Asp对Raji、Namalwa和CA46细胞株的作用，发现L-Asp诱导的BL细胞凋亡依赖PI3K/Akt/mTOR信号通路。

本研究发现，L-Asp浓度>0.1 U/ml时对3种BL细胞株的增殖有明显的抑制作用，但其抑制率在>1 U/ml的浓度下并不呈剂量依赖关系，而呈现出轻微时间依赖性。与He等[21]2014年在急性髓系白血病U937细胞中的研究结果相符。细胞周期调节蛋白异常导致肿瘤细胞的增殖不受控制，因此靶向细胞周期被认为是治疗肿瘤的有效方法[22]。本研究表明，L-Asp通过增加P21和P27的表达、降低Cyclin E的表达，将BL细胞株的细胞周期阻滞在G_0_/G_1_期，与Ueno等[23]证明的L-Asp诱导白血病细胞株L5178Y细胞周期停滞在G_1_期的研究结果一致。

L-Asp上调了死亡受体DR5 以及Caspase-8 的裂解活化，随后细胞凋亡程序的执行者Caspase-3激活，PARP切割失活，表明L-Asp诱导了外源性凋亡途径。与Song等[24]证明的L-Asp对慢性髓性白血病（CML）细胞株K562和KU812有明显的增殖抑制和诱导凋亡作用相符。此外，我们发现抗凋亡蛋白 Mcl-1和XIAP 的表达降低，促凋亡蛋白Bax、Bim、Noxa和cytochrome C的表达增加，表明L-Asp也激活了内源性凋亡途径。

肿瘤细胞生长需要门冬酰胺，然而其不能自身合成，需要从外界摄取。L-Asp催化血液中的门冬酰胺水解为门冬氨酸和氨，快速降低血浆中左旋门冬酰胺的含量，导致左旋门冬酰胺缺乏，从而选择性杀灭肿瘤细胞[25]。既往研究证实，氨基酸的枯竭可以诱导自噬[26]。在正常情况下，自噬水平一般较低，在缺氧、缺乏营养或化疗时，应激诱导的自噬激活与反复化疗耐药相关[27]。Song等[24]提出，L-Asp可以诱导CML细胞株K562和KU812的自噬反应。我们的实验结果证明，L-Asp可以诱导BL细胞株自噬，L-Asp浓度>1 U/ml时部分细胞出现耐药可能与自噬相关，但自噬与细胞存活、凋亡间的关系还需进一步研究[28]。

PI3K/Akt/mTOR途径的激活突变在人类癌症中经常发生，该途径的组成性激活促进细胞增殖和存活[29]。在本研究中，我们发现在L-Asp作用下，BL细胞株中PI3K、Akt-S473和mTOR的磷酸化水平以及mTOR磷酸化底物（p-p70S6K和p-4E-BP1）的表达降低，表明L-Asp抑制PI3K/Akt/mTOR通路。我们通过使用PI3K抑制剂LY294002联合L-Asp发现抑制PI3K磷酸化增加了细胞凋亡，提示PI3K/Akt/mTOR信号通路参与了L-Asp诱导的BL细胞凋亡。与此同时，Western blot结果显示，L-Asp处理组ERK及STAT3磷酸化水平降低，提示STAT3及MAPK相关信号通路可能也参与了L-Asp诱导的凋亡过程，与既往研究中提出的STAT3、ERK1/2信号途径可能成为BL治疗新靶点等结果一致[30]–[31]，但具体机制仍待继续研究。

综上所述，L-Asp明显抑制BL细胞株增殖，诱导G_0_/G_1_期周期阻滞和细胞自噬，激活内源和外源性细胞凋亡通路，同时L-Asp通过抑制PI3K/Akt/mTOR信号通路调控细胞凋亡。
